# Structural Covariance Network of Cortical Gyrification in Benign Childhood Epilepsy with Centrotemporal Spikes

**DOI:** 10.3389/fneur.2018.00010

**Published:** 2018-02-05

**Authors:** Lin Jiang, Tijiang Zhang, Fajin Lv, Shiguang Li, Heng Liu, Zhiwei Zhang, Tianyou Luo

**Affiliations:** ^1^Department of Radiology, The First Affiliated Hospital of Chongqing Medical University, Chongqing, China; ^2^Department of Radiology, The Third Affiliated Hospital of Zunyi Medical College, Zunyi, China; ^3^Department of Radiology, Affiliated Hospital of Zunyi Medical College, Medical Imaging Center of Guizhou Province, Zunyi, China

**Keywords:** Rolandic epilepsy, MRI, cortical development, connectome, hub

## Abstract

Benign childhood epilepsy with centrotemporal spikes (BECTS) is associated with cognitive and language problems. According to recent studies, disruptions in brain structure and function in children with BECTS are beyond a Rolandic focus, suggesting atypical cortical development. However, previous studies utilizing surface-based metrics (e.g., cortical gyrification) and their structural covariance networks at high resolution in children with BECTS are limited. Twenty-six children with BECTS (15 males/11 females; 10.35 ± 2.91 years) and 26 demographically matched controls (15 males/11 females; 11.35 ± 2.51 years) were included in this study and subjected to high-resolution structural brain MRI scans. The gyrification index was calculated, and structural brain networks were reconstructed based on the covariance of the cortical folding. In the BECTS group, significantly increased gyrification was observed in the bilateral Sylvain fissures and the left pars triangularis, temporal, rostral middle frontal, lateral orbitofrontal, and supramarginal areas (cluster-corrected *p* < 0.05). Global brain network measures were not significantly different between the groups; however, the nodal alterations were most pronounced in the insular, frontal, temporal, and occipital lobes (FDR corrected, *p* < 0.05). In children with BECTS, brain hubs increased in number and tended to shift to sensorimotor and temporal areas. Furthermore, we observed significantly positive relationships between the gyrification index and age (vertex *p* < 0.001, cluster-level correction) as well as duration of epilepsy (vertex *p* < 0.001, cluster-level correction). Our results suggest that BECTS may be a condition that features abnormal over-folding of the Sylvian fissures and uncoordinated development of structural wiring, disrupted nodal profiles of centrality, and shifted hub distribution, which potentially represents a neuroanatomical hallmark of BECTS in the developing brain.

## Introduction

Benign childhood epilepsy with centrotemporal spikes (BECTS) is one of the most common types of epilepsy occurring between the age of 3–13 years, typically characterized by a total of 2–10 infrequent seizures, resolution by the age of 15–17 years (1), an excellent prognosis, and a “benign” nature. BECTS children frequently have language, cognitive, and somatosensory problems after reaching adulthood (2). However, the neural basis for these problems is largely unclear.

Neuroimaging techniques have enabled improved characterization of the neural basis of BECTS. For example, morphometric studies revealed subtle anomalies in gray matter volume in the frontal, temporal, and left pars triangularis regions in BECTS children (3, 4). Diffusion-weighted studies have suggested the compromised microstructure of white matter in bilateral sensorimotor regions (5–7). Language task paradigms have also revealed atypical activation across different brain regions, particularly increased activation in the frontal, parietal and temporal areas (8–13). In addition, low-frequency fluctuations of spontaneous brain activity suggest abnormal local and global connections in BECTS patients (9, 14–24). Thus, children with BECTS present a wide distribution of alterations in the functional and structural organization within the brain.

Despite a number of existing neuroimaging studies, the etiology of BECTS remains elusive. BECTS is a unique age-dependent epilepsy with rare seizures, focal EEG abnormalities affecting the same well-delineated cortical region in most patients, and frequent mild to moderate cognitive dysfunctions. These problems, which are indicative of hemifacial seizures, as well as language and cognitive problems potentially stem from anomalous Sylvian fissure maturation. High-resolution structural imaging may facilitate exploration of the cortical landscape of BECTS. One recently validated algorithm, the local gyrification index, which detects subtle changes in the cortical landscape, permits the detection of atypical cortical folding in BECTS (25).

Recently, there have been increasing efforts to investigate the structural covariance of the coordinated cortical neuroanatomy (26–28). Such analysis will facilitate the qualification of anatomical relationships among cortical parcellations based on inter-areal covariation of different morphometric features, such as gray matter volume, cortical thickness, or even local gyrification. Although the biological significance of these association matrices remains vague, networks of gray matter covariance reflect patterns of coordinated structural maturation and disease propagation effects (28, 29). Based on associations between coordinated developmental changes in cortical networks and patterns of inter-areal covariations, children with BECTS likely exhibit differences in cortical folding (i.e., local gyrification) covariance, specifically reflecting the emergence of centrotemporal spikes during a critical period of brain development (i.e., from preschool to adolescence).

Therefore, in this study, we applied a graph theory analysis, the well-known connectome (30), of structural covariance based on the cortical gyrification index to characterize large-scale structural organization within the brain. This approach used graph theory to depict the human brain as a complex network comprising nodes (i.e., structural parcellations) and edges (i.e., structural covariance of the cortical gyrification) between the nodes. We investigated differences in cortical folding (i.e., local gyrification) and the covariance of the organization of networks of cortical folding patterns (i.e., local gyrification) between BECTS patients and their normally developing peer controls.

## Materials and Methods

### Participants

We recruited 26 BECTS children (15 males/11 females; mean age ± SD: 10.35 ± 2.91 years) and 26 demographically matched healthy controls (15 males/11 females; mean age ± SD: 11.35 ± 2.51 years) (Table 1). The inclusion criteria for BECTS subjects were (a) BECTS with partial or secondarily generalized tonic–clonic seizures diagnosed by a board-certified pediatric neurologist based on International League Against Epilepsy criteria, excluding children with multiple seizure types; (b) EEG within the past year showing identifiable centrotemporal sharp waves/spikes and epileptiform activity activated by sleep; (c) 1–10 seizures within the past year; and (d) normal physical/neurological examinations. Exclusion criteria were (a) a history of serious medical or psychiatric disorder and (b) an imaging study suggesting a progressive structural central nervous system lesion. All subjects were right-handed. The mean duration of epilepsy from onset to time of scanning was 20.7 months (SD = 20, min = 4, and max = 84). At the time of inclusion in this study, EEG spike foci were left-sided in 14 patients, right-sided in five patients and bilateral in seven patients. This study was approved through the Medical Ethics Committee of the Affiliated Hospital of Zunyi Medical College, and written informed consent was obtained from all participants or their guardians after a complete description of the required measurements. Detailed demographic information and clinical measures are listed in Table 1.

**Table 1:** Demographic details of the study cohorts.

Categories	BECTS (*n* = 26)	Controls (*n* = 26)	*p* Value
Sex (M/F)	15/11	15/11	>0.99
Age (years)	10.35 ± 2.91	11.35 ± 2.51	0.92
Education (years)	4.31 ± 2.77	5.65 ± 2.53	0.01
Handedness	26R	26R	>0.99
Duration (months)	Mean 38.81 (1–96)		–
IQ language	93.46 ± 14.73		–
IQ performance	85.77 ± 14.71		–
IQ over all	69.42 ± 9.52		–

### Neurobehavioral Assessments

A comprehensive battery of standardized neuropsychological tests and MRI were performed on the same day for children with BECTS, including verbal and non-verbal intelligence, verbal–auditory memory, visual processing speed, visual–spatial attention, and cognitive flexibility and inhibition, to assess the cognitive abilities of each subject. The Wechsler Intelligence Scale for Children (WISC-IV) and its four subscales (31) were used.

### MRI Acquisition

High-resolution structural MR images from all participants were collected using a GE 3.0-T (HDxt, GE Healthcare) scanner, stationed in the Department of the Radiology, the Affiliated Hospital of Zunyi Medical College, capturing T1-weighted 3D brain volume imaging (BRAVO)-sequence images (repetition time = 1,900 ms, echo times = 2.1 ms, inversion time = 900 ms, flip angle = 9°, slice thickness = 1.00 mm, and matrix size = 256 × 256) yielding 160 axial slices with an in-plane resolution of 1.0 mm × 1.0 mm.

### MRI Preprocessing

T1-weighted structural images were preprocessed using SPM12^1^ and Computational Anatomy Toolbox (CAT)^2^ based on MATLAB. Briefly, high-resolution structural images were first normalized to a customized child-sized brain template generated using the Template-O-Matic Toolbox^3^ and subsequently segmented into gray matter, white matter, and cerebrospinal fluid. Using a projection-based thickness procedure, the cortical thickness was estimated, and central cortical surfaces for both hemispheres were created (32).

### Calculation of Gyrification Parameters

The cortical thickness and central surfaces were employed as the basis to calculate gyrification index (33). We extracted the gyrification index based on absolute mean curvature, as described by Luders et al. (34). Briefly, cortical gyrification was established by calculating the mean curvature (35) across thousands of vertices on each individual central surface mesh model. The mean curvature maps (hereafter referred to as the gyrification index) were smoothed with a full width at a half maximum of 15 mm (36). The resulting smoothed cortical gyrification index was used for further statistical analyses and network construction.

### Construction of a Structural Covariance Network

Graph analysis was performed using the Graph-Theoretical Analysis Toolbox (GAT) (37). We defined nodes using the Destrieux Atlas in FreeSurfer (38) with 148 cortical regions of interest (ROIs), and we defined edges by determining the inter-regional Pearson’s correlation of cortical gyrification extracted from the individual gyrification surfaces in each group, with education included as covariates of nuisance.

We constructed a 148 × 148 association matrix *M* for each group with inter-regional Pearson’s correlation coefficients (*r_ij_*) between each pair of ROIs “*i*” and “*j*,” then thresholded the *r_ij_* into binary matrix *A* with values of 0 or 1, with thresholds setting across a range of network densities (*D*_min_–*D*_max_), where *D*_min_ (in this study, *D*_min_ = 0.11 and *D*_max_ = 0.45) means the lowest density that can make the networks fully connected (37). We computed both global and regional network measures across the density range of *D*_min_–*D*_max_ (i.e., 0.11–0.45) at an interval 0.02. Then, by considering matrix *A* to be graph G, we defined the quantities *N, K, D, i, j*, and *k* as the total number of ROIs (i.e., 148), number of edges, percentage of surviving edges at a specific density threshold, and three randomly selected nodes, respectively. For a detailed description, see Ref. (37).

### Global Network Properties

We calculated global metrics including clustering, path length, small-worldness, and global efficiency. These measures are well characterized in the network analyses, and have been extensively described elsewhere (39, 40). Briefly, the small-worldness was defined as a combination of high clustering and short path length, i.e., the ratio of normalized clustering and normalized path length, defined as follows (41):

$$\text{Small-worldness }\sigma =\frac{C/C_{\mathrm{rand}}}{L/L_{\mathrm{rand}}},$$

where *C* denotes the clustering coefficient, *L* represents the characteristic path length, and *C*_rand_ and *L*_rand_ are the mean *C* and *L* of 20 random networks, and σ means the ratio of normalized clustering and normalized path length (i.e., small-worldness) (41). For the small-worldness index, we calculated the measures both at *D*_min_ (0.11) as well as across a range of densities (0.11–0.45) using the area under the curve (AUC). A network was considered small-world when the ratio σ at *D*_min_ was >1 (41).

### Regional Network Properties and Hub Identification

We also computed regional centrality measures, including betweenness *b_i_*, normalized degree *k_i_*, and hubs’ identification based on the *b_i_* and *k_i_*, then quantified the within- and between-group differences:

$$b_{i}=\sum_{m\neq i\neq n\in G}\frac{\sigma_{mn}(i)}{\sigma_{mn}},$$

$$k_{i}^{B}=\sum_{\;j\in G}a_{ij}\text{ or}$$

$$k_{i}^{W}=\sum_{j\in G}W_{ij},$$

where *b_i_* denotes the sum of the shortest paths that pass-through node *i, k_i_* represents the number of edges between node i and other nodes, and *b* and *k* represent the mean betweenness and degree, respectively, of the entire network. Furthermore, *b_i_* and *k_i_* were used to identify the hubs of the network; nodes with regional values of at least 2 SDs greater than the mean value were recognized as hubs.

### Statistics

For the cortical gyrification maps, we performed vertex-wise group inference on the smoothed cortical surfaces using general linear modeling implemented in CAT (40, 42), these including (1) vertex-wise gyrification differences between patients and controls, (2) correlations with neurobehavioral measures, and (3) correlations with chronological age (development). The results were corrected for multiple comparisons using a Monte Carlo simulation, with 10,000 iterations (family-wise error). For the topological measures, within- and between-group comparisons were done by using the GAT toolbox.

## Results

Table 1 shows the demographics for all subjects. There were no statistically significant differences between BECTS children and controls in relation to age (*p* = 0.92), gender (*p* > 0.99), and handedness (*p* > 0.99), although education showed significant differences between groups (*p* = 0.01) (Table 1).

### Between-Group Analysis of Cortical Gyrification

**Figure 1:**
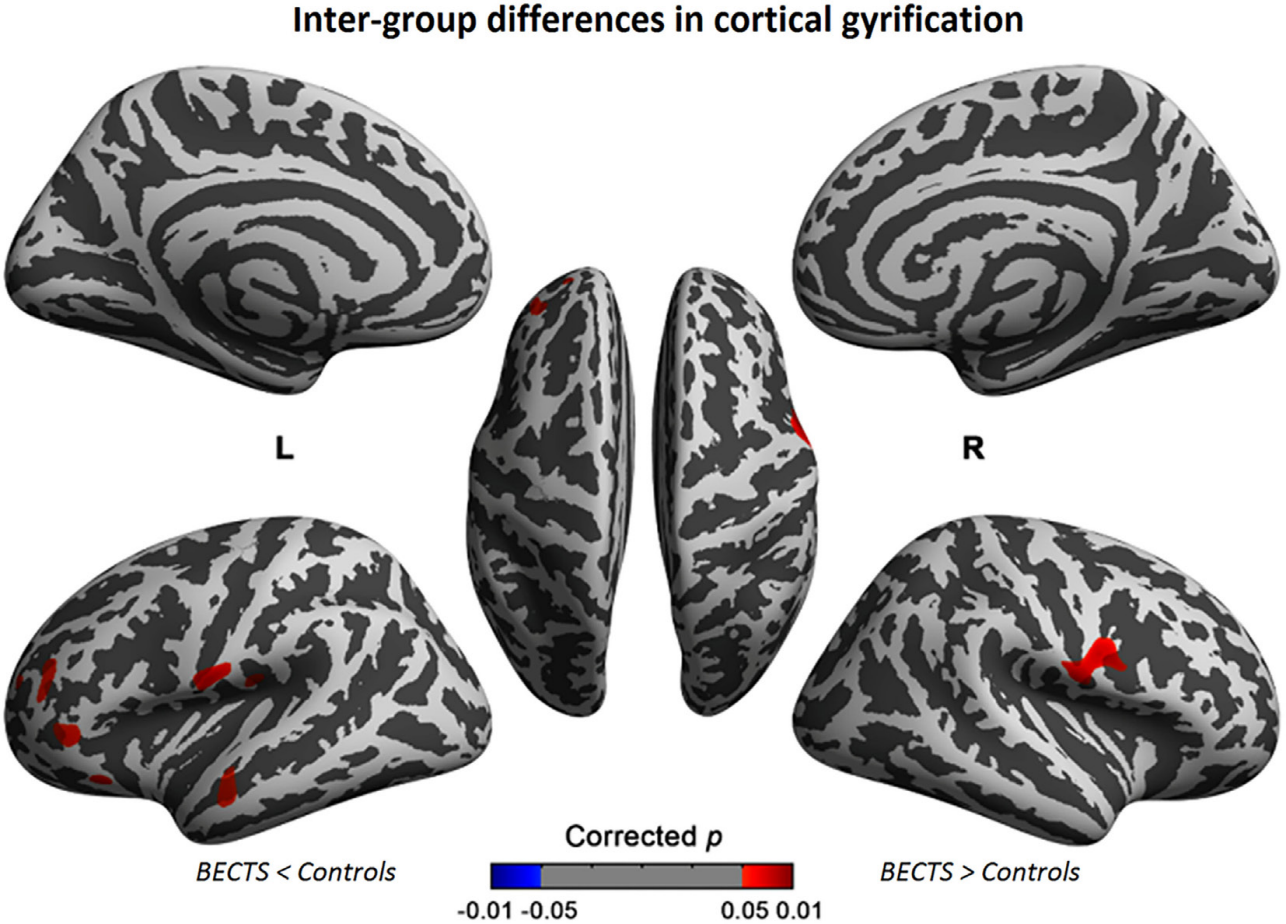
Significant cortical gyrification clusters projected onto the inflated surfaces of the bilateral hemispheres. The significant clusters show increased gyrification in the BECTS group compared with their peer controls and surviving cluster correction (*p* < 0.05). Detailed information for these clusters is shown in Table 2.

The cortical gyrification analysis identified several clusters in bilateral cerebral cortices that were increased as the BECTS group compared with the controls (Figure 1; Table 2). These clusters included significantly increased gyrification in the bilateral Sylvain fissures, left pars triangularis, temporal, rostral middle frontal, lateral orbitofrontal, and supramarginal areas (Figure 1; Table 2). No significantly decreased clusters survived whole brain correction.

**Table 2:** Results of group comparison of cortical gyrification between BECTS and controls groups.

Left patient > control			Right patient > control		
Surface area	Vertex	*p* Value	Surface area	Vertex	*p* Value
Sylvan fissure	539	0.00273*	Sylvan fissure	1,022	0.00273*
Par striangularis	263	0.00636*			
Temporal	254	0.00818*			
Rostralmiddle frontal	235	0.00818*			
Lateralorbito frontal	129	0.00636*			
Supramarginal	47	0.00909*			

*Based on Desikan-Killiany DK40 atlas.

### Within-Group Global Network Measures

**Figure 2:**
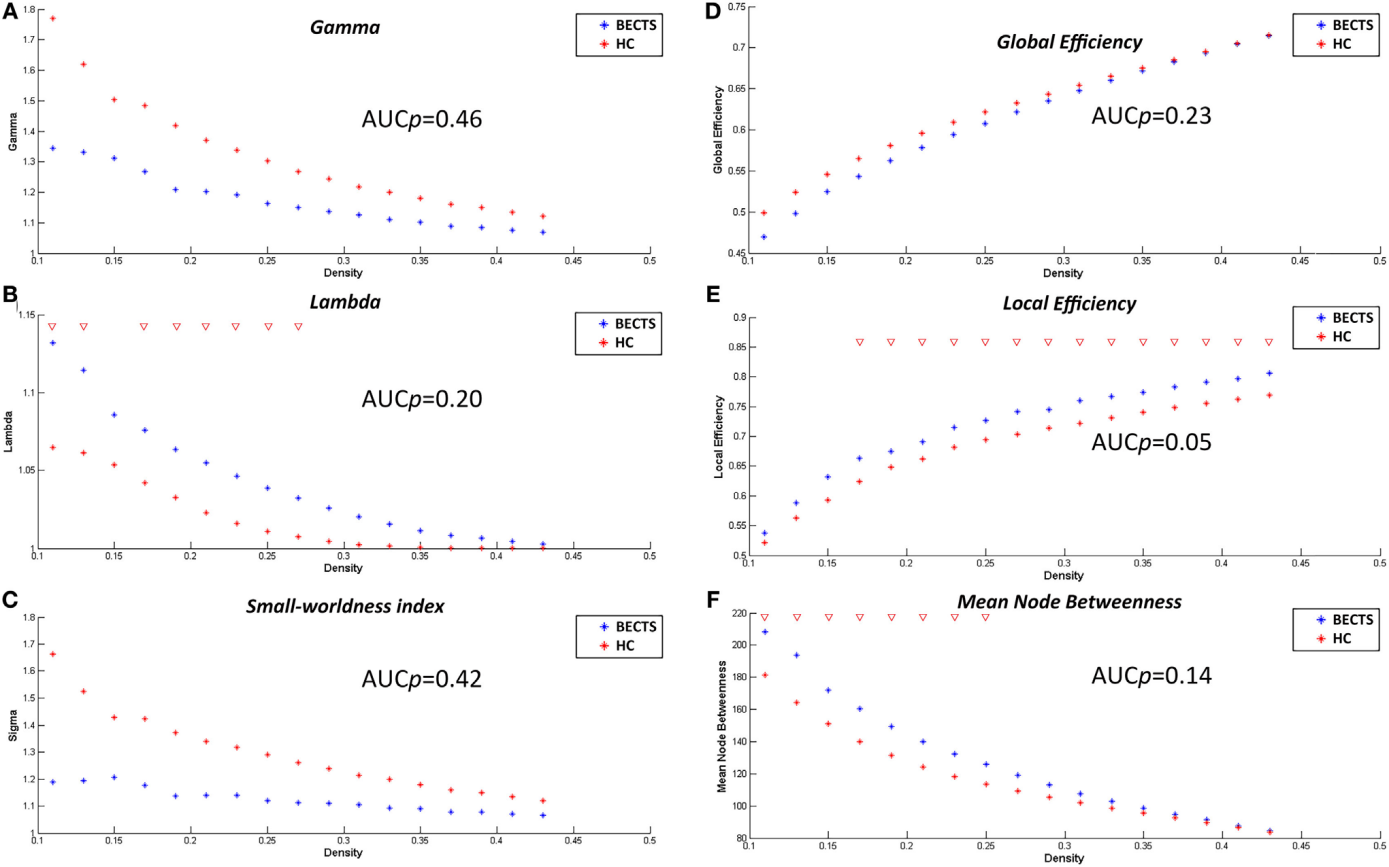
Within-group global network measures, between-group differences in these measures across a range of network densities, and area under the curve (AUC) results. Normalized clustering **(A)**, normalized path length **(B)**, small-world index **(C)**, global efficiency **(D)**, local efficiency **(E)**, and mean node betweenness **(F)** of the BECTS and healthy control (HC) networks. The red inverted triangle indicates the difference between the two groups.

The lowest network density that made the networks fully connected was at *D*_min_ = 0.11 in this study. To explore alterations in the network topology as a function of network density, we thresholded the constructed association matrices at the range of 0.11–0.45, with an interval of 0.02. Changes in the global measures as a function of network density are shown in Figure 2. The networks of both groups complied with a small-world organization, i.e., higher clustering than as well as short length path close to the random networks. That is, normalized clustering greater than 1 and a normalized path length close to 1. These metrics are thresholded both at the *D*_min_ (0.11), and across the predefined network densities interval (0.11–0.45).

### Between-Group Differences in Global Network Measures

#### Differences across Network Densities

We examined the inter-group differences in the global metrics across the predefined network densities (0.11:0.02:0.45) (Figure 2). Both groups showed a small-world organization, i.e., a combination of higher clustering as well as short length path as mentioned earlier. Although the BECTS group showed a lower normalized clustering and a higher normalized path length, and a less optimal small-worldness, there were no significant inter-group differences (*p* > 0.05), suggesting a less optimized network architecture for children with BECTS.

#### AUC Analysis of Global Network Measures

In addition to contrasting the global measures at various densities, we compared their AUC (density range of 0.11:0.02:0.45) between the two groups. Similar to the case of different densities, the BECTS group did not exhibit a significantly different AUC for global network measures (*p*_gamma = 0.46, *p*_lambda = 0.20, *p*_sigma = 0.42, *p*_global efficiency = 0.23, *p*_mean local efficiency = 0.05, and *p*_mean node betweenness = 0.14) compared with the controls (Figure 2).

### Between-Group Differences in Regional Network Measures

We also compared the regional metrics (at minimal network density range of 0.11) between the two groups (Figures S1A–C in Supplementary Material). Regions of normalized degree centrality, including the left middle occipital gyrus, left parahippocampal gyrus, and the inferior segment of the left circular sulcus of the insula, were significantly greater for the BECTS group, while the right middle frontal sulcus exhibited a significantly smaller degree centrality in the BECTS group (Figure S1A in Supplementary Material). Among regions of normalized nodal betweenness centrality, only the left triangular part of the inferior frontal gyrus and the inferior segment of the circular sulcus of the insula exhibited significantly greater betweenness in the BECTS group (Figure S1B in Supplementary Material). Among regions of normalized regional clustering, only the right transverse temporal sulcus exhibited significantly smaller clustering in the BECTS group (Figure S1C in Supplementary Material). All aforementioned regions survived following FDR correction (*p* < 0.05).

### AUC Analysis for Regional Measures

**Figure 3:**
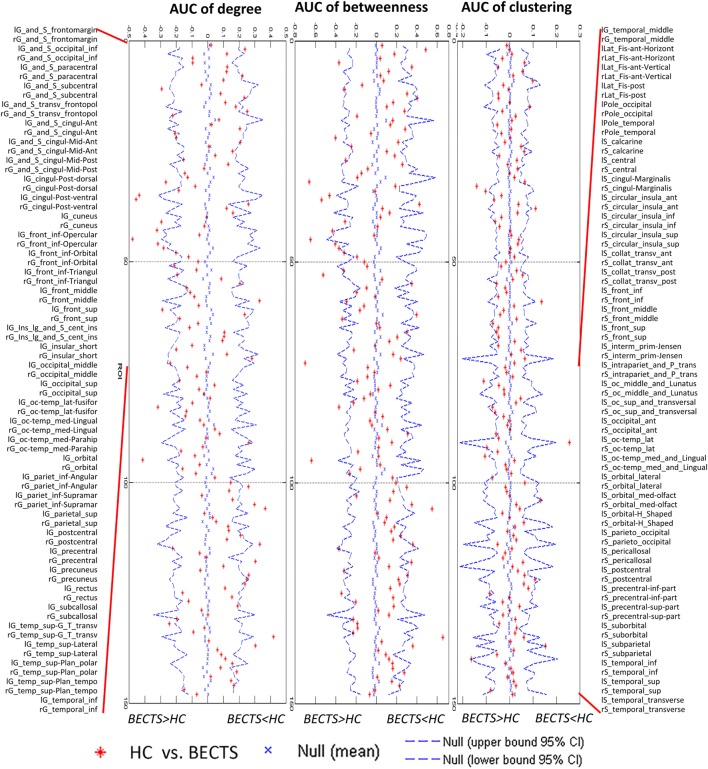
Between-group differences of regional measures (normalized regional degree, betweenness, and clustering) across a range of network densities [i.e., area under the curve (AUC) results]. The red inverted triangle indicates the difference between the two groups. All regions survived following FDR correction (*p* < 0.05).

For the AUC of the regional network measure curves between the groups, the BECTS group showed increased normalized nodal clustering in the long insular gyrus and central sulcus of the left insula, similar to the identified differences across densities (Figure 3). The BECTS group also showed a greater normalized regional degree in several regions, including the right short insular gyri, left middle occipital gyrus, and left parahippocampal gyrus, and smaller degree centrality in the right middle frontal and left precentral sulcus (Figure 3). For normalized regional betweenness, the BECTS group showed greater betweenness centrality in the left inferior temporal gyrus and smaller betweenness centrality in the right middle frontal sulcus and inferior part of the left precentral sulcus (Figure 3). All aforementioned regions survived following FDR correction (*p* < 0.05).

### Network Hubs

**Figure 4:**
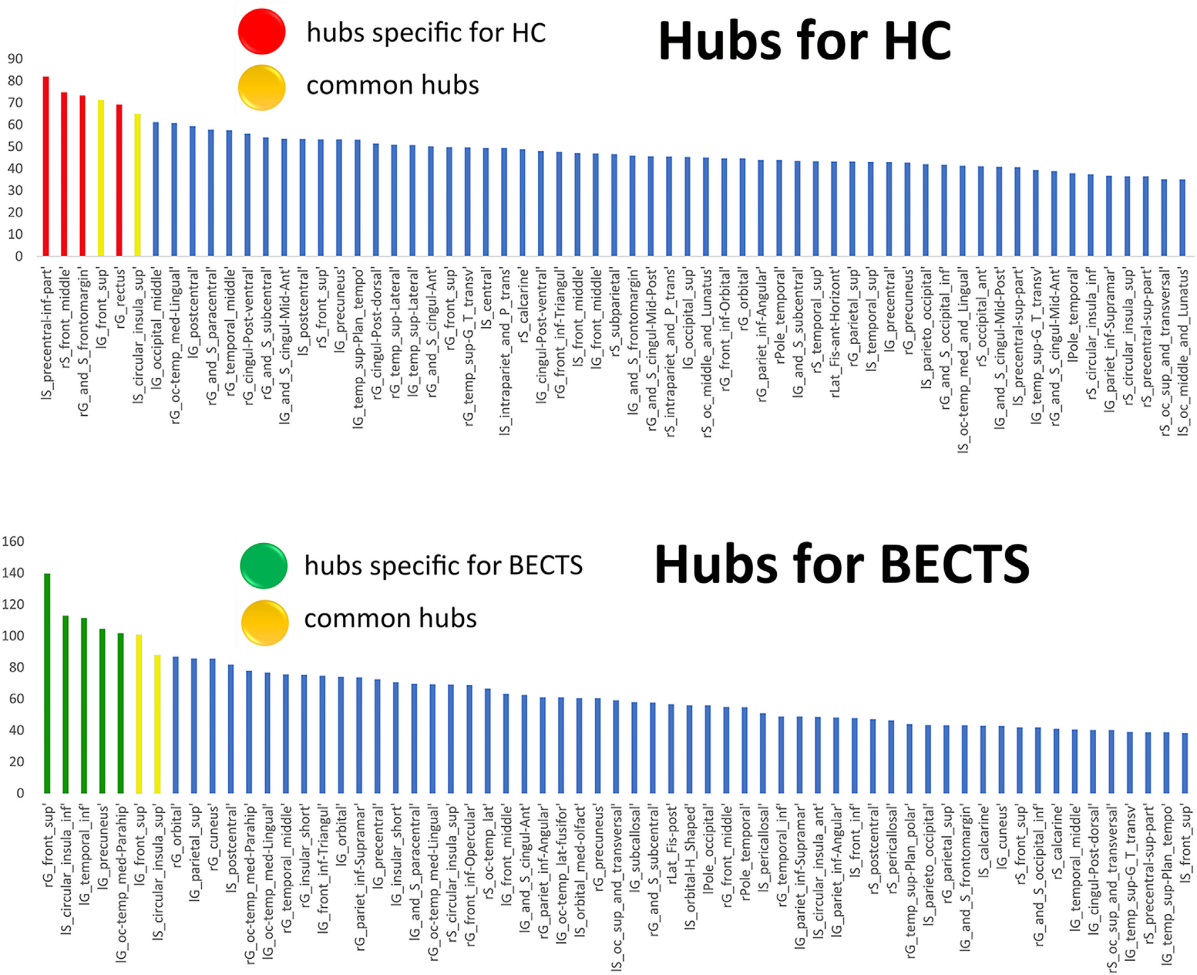
Network hubs. The red color indicates hubs specific to healthy controls (HCs), the green color highlights hubs specific to children with BECTS, and the yellow color represents hubs that are common in both groups.

We used regional betweenness centrality as the basis for hub classification. A hub node was defined as the detected regional betweenness centrality 2 SDs higher than the mean network betweenness. Hubs were calculated based on the AUC of the nodal betweenness across a range of network densities (density range of 0.11:0.02:0.45) (Figure 4; Figure S2 and Table S1 in Supplementary Material for detailed results). The common hubs in both groups included the left superior frontal gyrus and left inferior segment of the circular sulcus of the insula. Network hubs specific for the BECTS group were identified in the right superior frontal gyrus, left parahippocampal gyrus, left precuneus, and left inferior temporal gyrus. Network hubs specific to controls were identified in the right fronto-marginal gyrus (of Wernicke) and sulcus, right straight gyrus, right middle frontal sulcus, and left inferior part of the precentral sulcus. The cores of the patient networks exhibited pattern changes characterized by a displacement of hubs to the left temporal and occipital lobes and a reduction in the right frontal lobe.

### Random Failure and Targeted Attack Analysis

**Figure 5:**
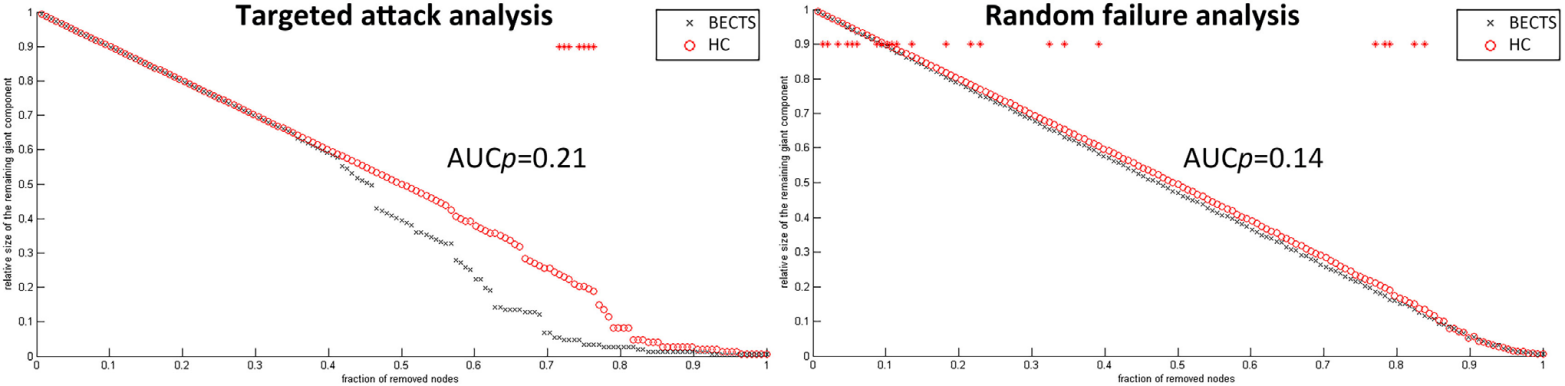
Random and targeted attack analysis. Alterations in the size of the largest preserving component of the network are shown as a function of a fraction of randomly (left panel) and targetedly (right panel) removed nodes. In many proportions of the deleted nodes and the area under the curve (AUC) results, there was no significant difference between the two groups (*p* > 0.05) of the resilience of the networks to both targeted and random attacks. In general, network of BECTS showed less robust to the targeted attack as compared with the controls, but the statistical significance only appears at a few fractions of deleted nodes (*p* < 0.05, the red star indicates a significant difference across different network densities between the two groups).

To analyze the vulnerability of the structural covariance networks to random as well as targeted attacks, we computed the mass of the largest persevering component in response to the successive removal of nodes randomly or targetedly. In all proportions of the deleted nodes, the resilience of the both structural networks to random failure was not significant different (*p* > 0.05) (left panel in Figure 5). Although the AUC of the resilience to random failure was increased in the BECTS group, this effect was not statistically significant (*p* value of AUC for target attack = 0.21, *p* value of AUC for random attack = 0.14). By contrast, the network of BECTS was less robust to targeted attack as compared with the control network, and this difference was significant for several fractions of attacked nodes (*p* < 0.05) (right panel in Figure 5). The same procedure was applied to analyze the response of the network to targeted attack by removing the nodes in a rank order of decreasing nodal betweenness centrality (right).

### Correlation Analyses

**Figure 6:**
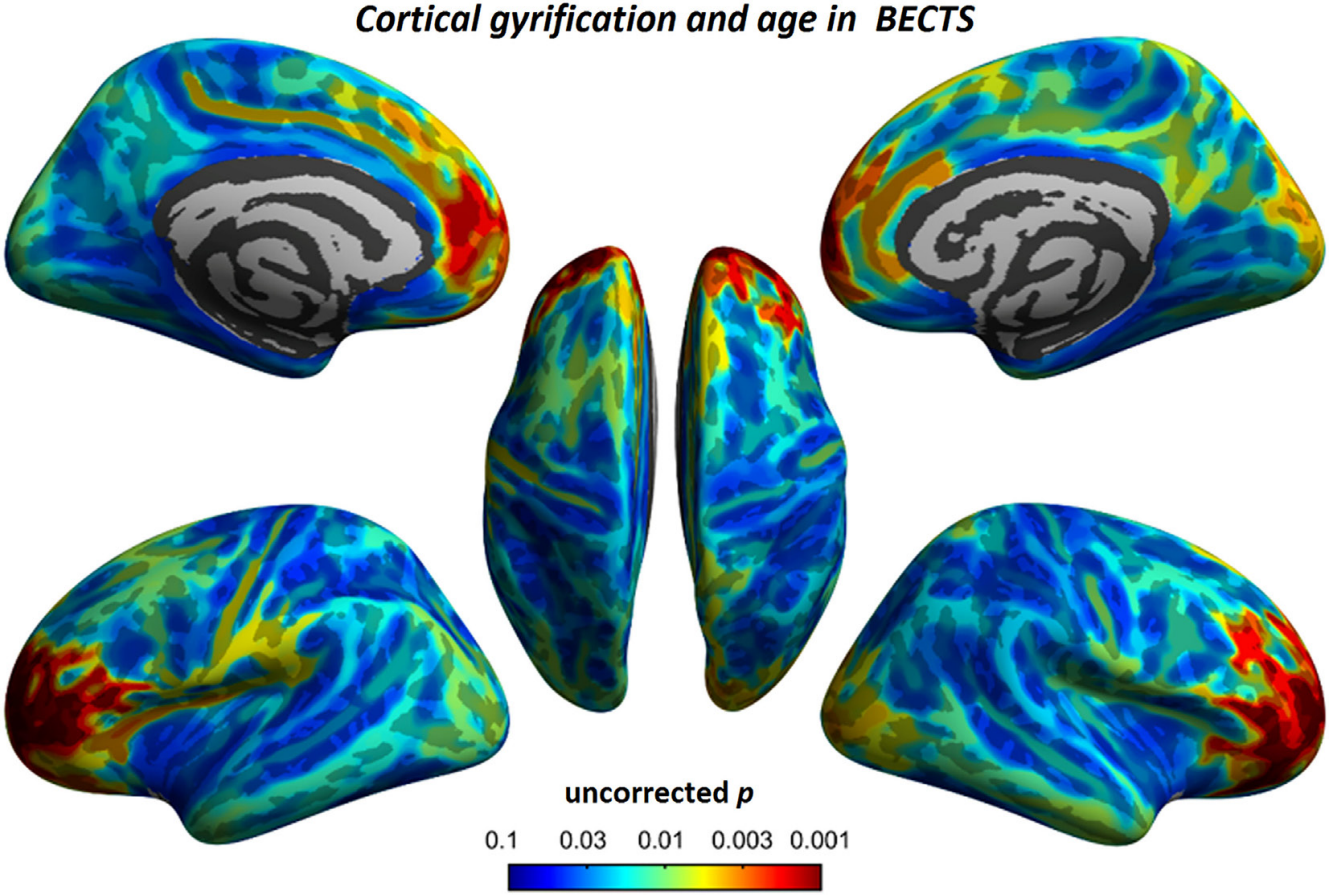
Relationship between cortical gyrification and chronological age in children with BECTS. In children with BECTS, we did not observe significant age effects on the foci of Sylvian fissures, but we did detect frontal association cortices with significant positive relationships between cortical gyrification and age (vertex *p* < 0.001, cluster-level correction).

**Figure 7:**
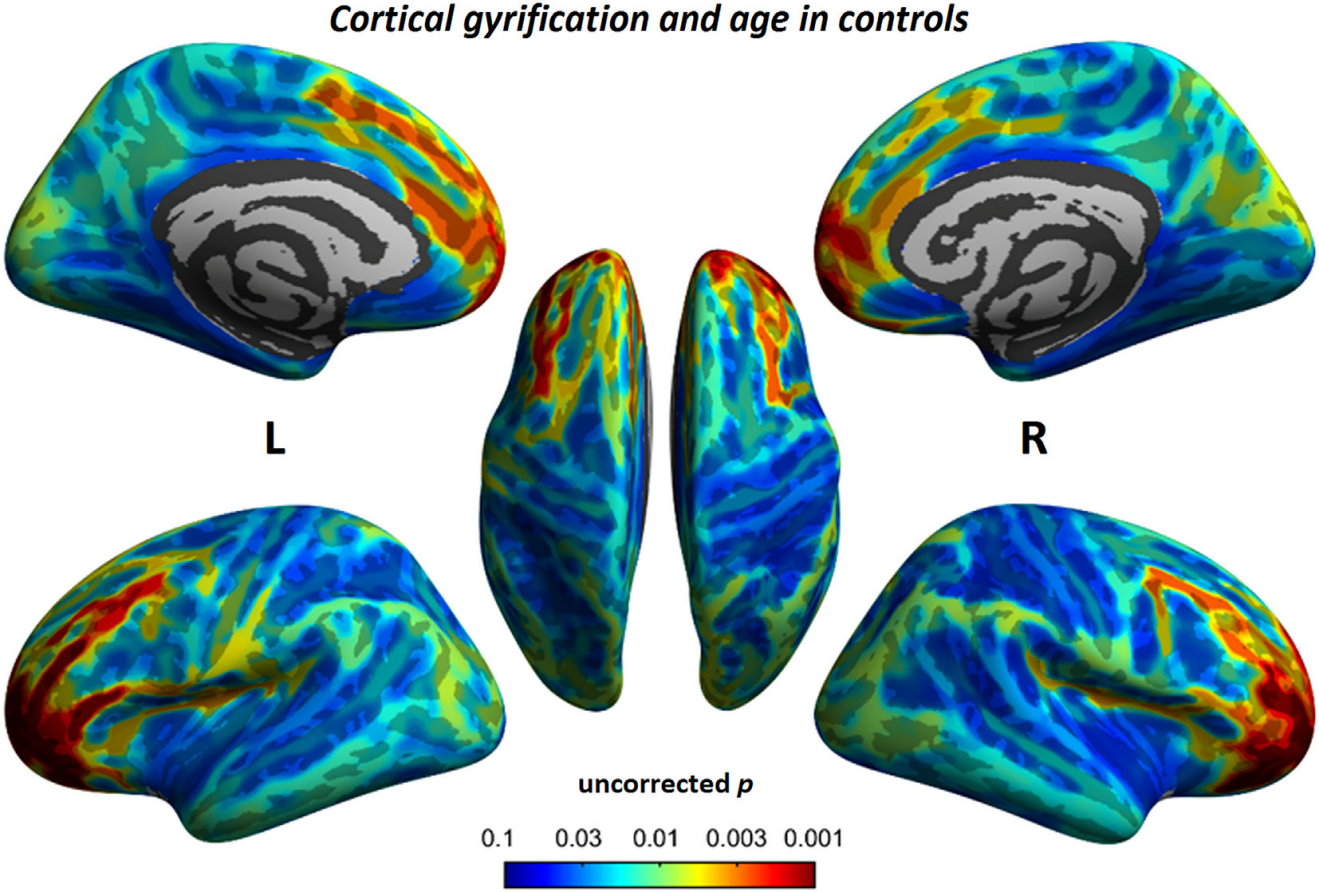
Relationship between cortical gyrification and chronological age in typical developmental controls. Similar to children with BECTS, we also observed significant positive relationships between cortical gyrification and age (vertex *p* < 0.001, cluster-level correction).

**Figure 8:**
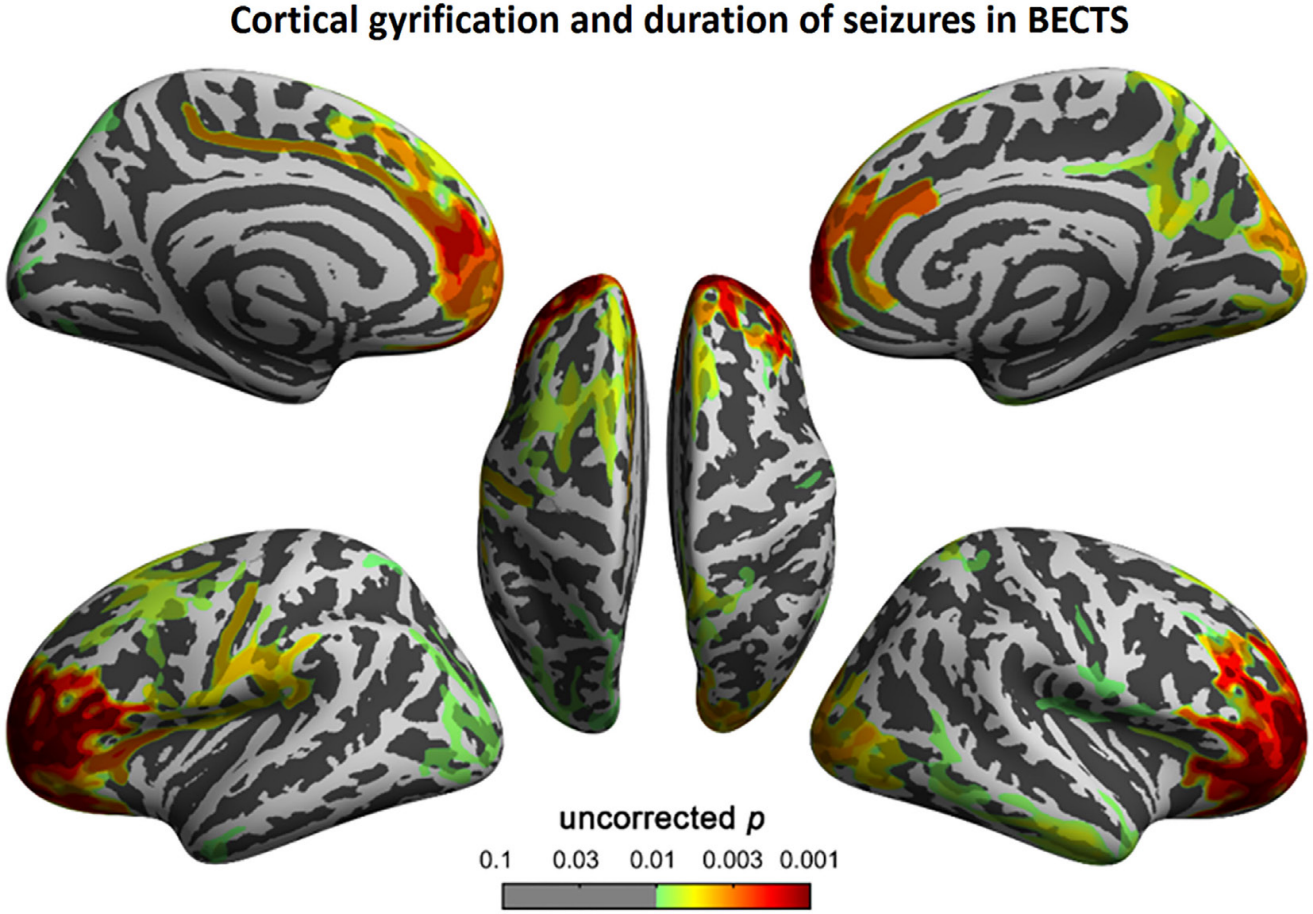
Correlation between cortical gyrification and duration (months) of epilepsy in children with BECTS. We observed a positive correlation between cortical gyrification in children with BECTS and the duration of epilepsy in the frontal cortices (vertex *p* < 0.001, cluster-level correction).

To understand the relationship between age and altered cortical gyrification in children with BECTS, it is instructive to perform a regression analysis with chronological age in children with BECTS and their peer controls. Although we did not observe significant effects of age on the foci of the bilateral Sylvian fissures, we did detect other foci with significant positive relationships between the gyrification index and age (vertex *p* < 0.001, cluster-level correction), suggesting the uncoordinated development of patterns of transmodal areas (Figures 6 and 7). The Sylvian fissures are highly variable transmodal structures. To explore whether children with BECTS have developmental problems, we further conducted multiple regressions ruling out potential developmental problems. We also observed positive relationships between the gyrification index and duration of epilepsy (vertex *p* < 0.001, cluster-level correction) (Figure 8).

## Discussion

We investigated differences in cortical folding and its structural covariance networks between children with BECTS and their peer controls to confirm the effects of centrotemporal spikes on the developing brain. Specifically, children with BECTS exhibited (1) aberrant foci of cortical gyrification in BECTS, including bilateral Sylvain’s fissures, left pars triangularis, temporal, rostral middle frontal, lateral orbitofrontal, and supramarginal areas, suggesting the preexistence of over-folding cortical sheets and subsequent atypical development of higher association cortices and (2) disrupted node properties in the structural network and a shift in the hub distribution. This study is the first to use a graph theory to investigate alterations in local gyrification covariance networks between BECTS adolescents and matched controls. Based on these findings, abnormal cortical folding and its nodal properties of structural wiring may underlie the neuroanatomical basis of BECTS.

The between-group results of cortical gyrification are generally in line with those of previous neuroimaging studies. The bilaterally increased gyrification in the Sylvian fissures was consistent with cortical thickness (14) and volumetric changes of gray matter (43), aberrant language recruitment using task paradigms (11), and altered functional connectivity and local intrinsic brain activity measured using resting-state fMRI in previous studies (10, 12, 15–19, 21–24, 44). For example, as shown in recent morphometric studies, children with BECTS have a thinner cortex in the frontal, temporal, and occipital regions and exhibit sparse (atypical) maturation of cortical thickness during development (14) as well as increased gray matter in the striatum and fronto–temporo–parietal cortex (striato–cortical circuit) (3, 43). In diffusion-weighted white matter integrity and anatomical connectivity analyses, BECTS patients demonstrated white matter impairment, particularly in the corpus callosum and forceps minor (5), the left superior longitudinal fasciculus, the retrolenticular region of the internal capsule, posterior thalamic radiation, and the sagittal stratum compared with controls (7). When considering a potential neurodevelopmental interpretation of these results, we observed a significant increase in the gyrification of Sylvian fissures. Anatomically, the Sylvian fissure comprises cytoarchitectonically discrete areas formed by the infolding or uneven growth of the outer cortex relative to inner structures of the frontal, parietal, and temporal opercula over the insula. Clinically, anomalous Sylvian fissure morphology was also observed in Williams syndrome (44, 45). This anatomical feature facilitates great morphological variation among hemispheres, both between and within brains. Altered cortical folding may result from neuronal or axonal injury. Alternatively, the folding abnormalities may share a common etiology, such as genetics, with BECTS. Apart from the Sylvian fissures, we also observed increased gyrification in the transmodal cortices, including the left pars triangularis, temporal, rostral middle frontal, lateral orbitofrontal, and supramarginal areas. The etiology of BECTS consists of centrotemporal spikes that lead to language, cognitive, and developmental dysfunction *via* perturbation of the underlying connectivity. Neuroimaging and lesion behavior studies have correlated Sylvian fissures with language development and linguistic behaviors. The orbitofrontal region plays a role in behavior and emotion, the pars triangularis area of language and executive functions, and the temporal–parietal regions in multidomain information processing. The increased cortical folding in these regions may provide biological insights associated with subsequent outcomes.

Beyond the comparison of local gyrification profiles, we must consider the network architecture of the gyrification of the brain anatomical covariance. Globally, BECTS patients showed an insignificantly different but less optimal connectome compared with the controls. These connections with decreased normalized local clustering, increased normalized path length, and reduced small-worldness, suggesting a deviation from optimal tradeoff between segregation and integration (28, 30, 41). Using various morphological measures to construct the covariance network, previous studies have stably demonstrated small-world architecture in structural brain networks during normal development in healthy subjects (46). This network organization enables efficient information processing by providing an optimal balance between segregation and integration. Despite the lack of significant inter-group differences, these measures were lower than previously reported.

We identified between-group differences in regional network measures across several brain systems, consistent with the between-group comparison in this study and previous findings. Several regions in the left occipital, left temporal, and left insular surfaces exhibited increased centrality (clustering, betweenness, and degree centrality), while other regions in the right middle frontal sulcus and left precentral sulcus showed reduced centrality in the BECTS network compared with the controls. As elements of brain networks, individual nodes have unique centralities crucial for defining functional specialization (30). Numerous neuropsychological studies have demonstrated subtle long-term language and neurocognitive deficits, specifically in cognitive functions subserved by the prefrontal, temporal, and sensorimotor cortices, in BECTS individuals (24). Some regions with increased centrality in the BECTS network are consistent with the between-group comparison of local gyrification in this study and have previously been demonstrated to have compromised gray matter volume, blood flow or neuronal integrity in individuals with BECTS (1–15). These studies may explain the enhanced centrality in the responding areas for fine motor and multidimensional sensory processing (especially the left side) and the reduced centrality in the prefrontal regions (particularly the right side) in the BECTS group, suggesting disrupted anatomical interactions among the frontal, insular, and temporal regions and the remaining areas of the brain.

The regions identified as hubs in both groups were involved in working memory, language, attention, and interoceptive functions. By contrast, the regions with increased centrality in BECTS were involved in memory, visuospatial processing, and self-awareness. For example, the anterior insula contains an interoceptive representation that provides the basis for all subjective feelings from the body (16). The anterior insula is often activated in conjunction with the anterior cingulate cortex, and these two structures function together as limbic sensory and motor cortices that engender salient feelings and motivations, respectively (17). In addition, we determined that the shared hubs between the two groups were limited to the left superior frontal gyrus and left inferior segment of the circular sulcus of the insula. We identified an association between increased hub number with a shift to non-hub regions during normal development in the BECTS group. This finding may correlate with the variability across individuals.

For random and targeted attack analysis, there was no statistical significance on the AUC between the two groups, suggesting a similar resilience of both networks in response to random failure and targeted attack. However, BECTS network showed less resilient in response to targeted attack, and the overall between-group difference was significant at several fractions of removing nodes. This observation is consistent with the results of the regional measures, suggesting the BECTS network has fewer central hubs than the controls because hubs in the structural connectome are energy demanding and vulnerable to major diseases (47).

BECTS occurs during a critical period of brain development and has been associated with subsequent language and cognitive issues. One study examined abnormalities in structural and functional connectivity and their convergence in this cohort. Previous studies showed significantly decreased structural/functional connectivity coupling in these children compared with their peer controls, with prominent impairment in centrotemporal network convergence (9). Over the past decade, there has been increasing interest in investigating the intrinsic brain architecture in BECTS using resting-state fMRI (48). This intrinsic architecture represents the topographies of functionally connected areas across the brain (also known as resting-state networks, or intrinsic connectivity networks) (48). Evidence for the impact of Rolandic spikes on the intrinsic architecture has revealed significant disruptions, both locally and globally. For example, Tang et al. (49) examined the regional homogeneity of resting blood-oxygen-level-dependent signals and observed increased regional homogeneity (local connectivity) in the central, premotor, and prefrontal regions and decreased local connectivity in bilateral orbitofrontal cortices and temporal poles in children with BECTS (49). Additional studies (21, 22) also investigated the regional homogeneity changes in BECTS children who were and were not receiving antiepileptic drug medications, showing that antiepileptic drug medications inhibit regional homogeneity in the epileptogenic focus. Other studies utilizing functional connectivity reported decreased default mode functionality (12) in children with BECTS, and this decreased functionality coupled with the frontoparietal network (15) to decrease inter-hemispheric functional connectivity between the bilateral frontal cortices and cerebellum (16), disrupt topological organization with reduced local segregation in sensorimotor areas, and decrease nodal centralities predominantly around the Rolandic fissure and other linguistics and attention control areas (19). More recently, a study using real-time EEG-fMRI successfully identified disruptions among the intrinsic networks subserving language, behavior, and cognition functions during interictal Rolandic spikes (or centrotemporal spikes) in children with BECTS (18). Such findings suggest Rolandic spikes disturb intrinsic activity, and these patterns may be reconfigured using antiepileptic medications.

There are several limitations to this study. First, although neurobehavioral assessments in normally developing controls were absent, we propose that these individuals had better profiles. Second, because of the limited sample size, we did not subdivide the patient cohort into different subgroups based on epileptogenic foci.

## Conclusion

This study is the first to apply a graph theory to investigate changes in local gyrification correlation networks between children with BECTS and matched controls. The results suggest BECTS may be a condition that features the abnormal over-folding of the Sylvian fissures and the uncoordinated development of structural wiring, disrupted nodal profiles of centrality, and shifted hub distribution that may represent a neuroanatomical hallmark of BECTS in the developing brain.

## Ethics Statement

This study was approved through the Medical Ethics Committee of the Affiliated Hospital of Zunyi Medical College, and written informed consent was obtained from all participants or their guardians after a complete description of the required measurements.

## Author Contributions

All authors listed have made a substantial, direct, and intellectual contribution to the work and approved it for publication.

## Conflict of Interest Statement

The authors declare that the research was conducted in the absence of any commercial or financial relationships that could be construed as a potential conflict of interest.

## Footnotes

^1^www.fil.ion.ucl.ac.uk/spm.

^2^http://www.neuro.uni-jena.de/cat/.

^3^http://dbm.neuro.uni-jena.de/software/tom/.

## Supplementary Material

Figure S1 Between-group differences of regional measures of normalized regional degree (A), betweenness (B), and clustering (C), and across a range of network densities [i.e., area under the curve (AUC) results] with corresponding measures in (D–F). The red inverted triangle indicates the difference between the two groups. All regions survived following FDR correction (p < 0.05).

Figure S2 Network hubs. The red color indicates hubs specific to healthy controls (HCs), the green color highlights hubs specific to children with Benign childhood epilepsy with centrotemporal spikes (BECTS), and the yellow color represents hubs that are common in both groups.
