# Supplementary figures and images for: Structural Covariance Network of Cortical Gyrification in Benign Childhood Epilepsy with Centrotemporal Spikes
Source: Front Neurol. 2018 Feb 5;9:10. doi: 10.3389/fneur.2018.00010 (PMC5807981; doi:10.3389/fneur.2018.00010)

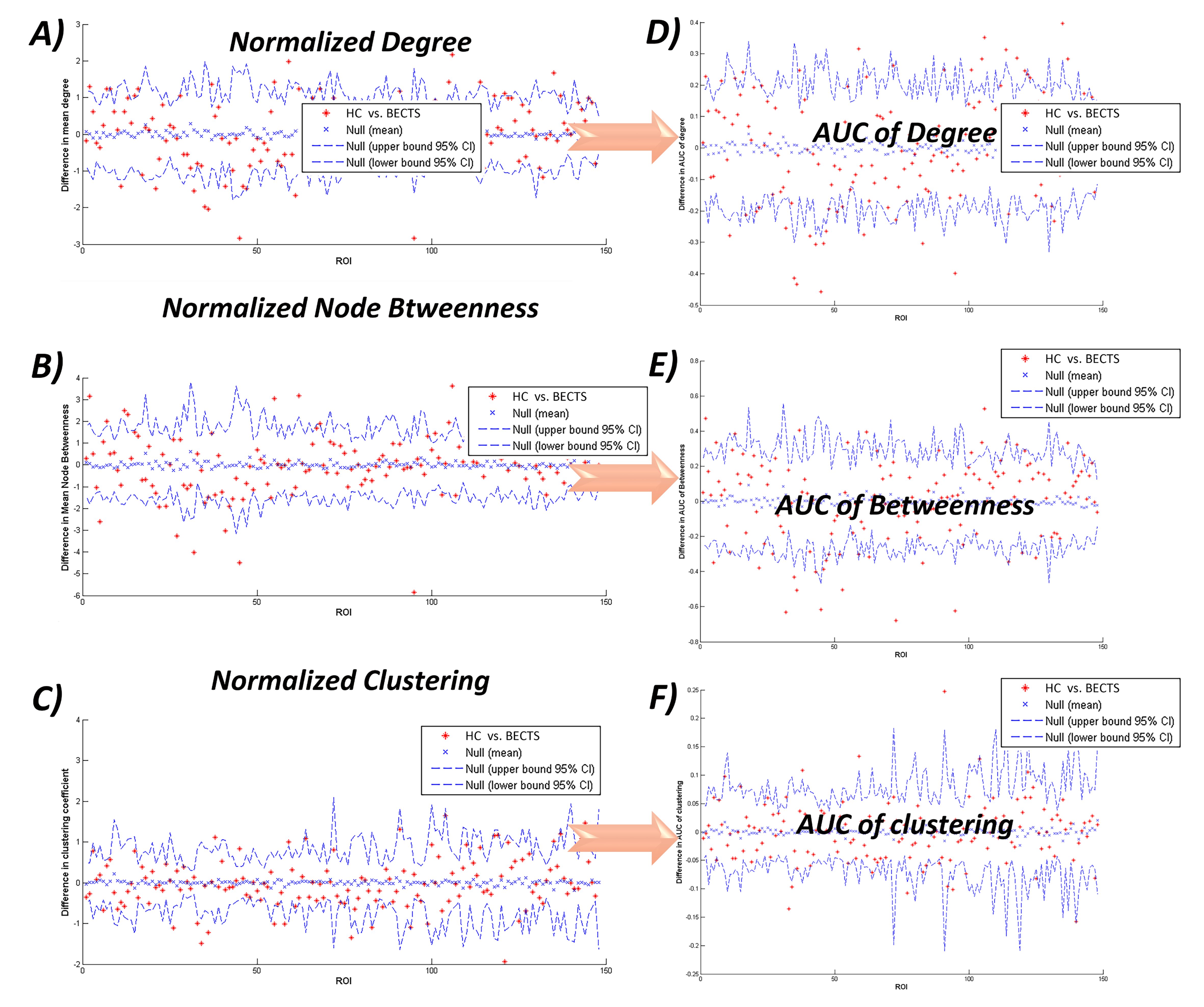

Supplement: Figure S1 — Between-group differences of regional measures of normalized regional degree (A), betweenness (B), and clustering (C), and across a range of network densities [i.e., area under the curve (AUC) results] with corresponding measures in (D–F). The red inverted triangle indicates the difference between the two groups. All regions survived following FDR correction (p < 0.05). [file Image_1.tif]

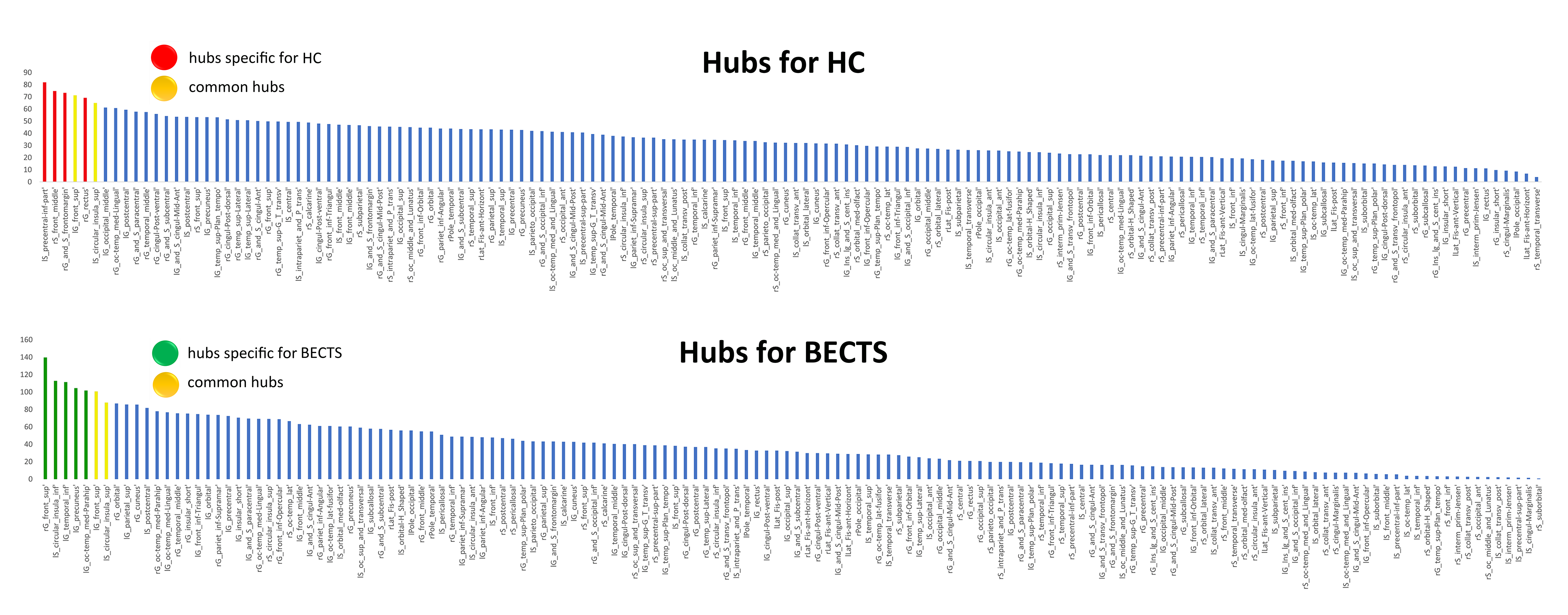

Supplement: Figure S2 — Network hubs. The red color indicates hubs specific to healthy controls (HCs), the green color highlights hubs specific to children with Benign childhood epilepsy with centrotemporal spikes (BECTS), and the yellow color represents hubs that are common in both groups. [file Image_2.tif]
